# Supplementary material for: Identification of Dietary Phytochemicals Capable of Enhancing the Autophagy Flux in HeLa and Caco-2 Human Cell Lines
Source: Antioxidants (Basel). 2020 Nov 27;9(12):1193. doi: 10.3390/antiox9121193 (PMC7760668; doi:10.3390/antiox9121193)
Supplement: Supplementary file 1 [file antioxidants-09-01193-s001.pdf]

antioxidants

Supplementary Materials

Identification of dietary phytochemicals capable of enhancing the autophagy flux in HeLa and Caco-2 human cell lines

Kohta Ohnishi[1,*], Satoshi Yano[2,‡], Moe Fujimoto[1,‡], Maiko Sakai[1], Erika Harumoto[1], Airi Furuichi[1], Masashi Masuda[1], Hirokazu Ohminami[1], Hisami Yamanaka-Okumura[1], Taichi Hara[2,*], Yutaka Taketani[1,*]

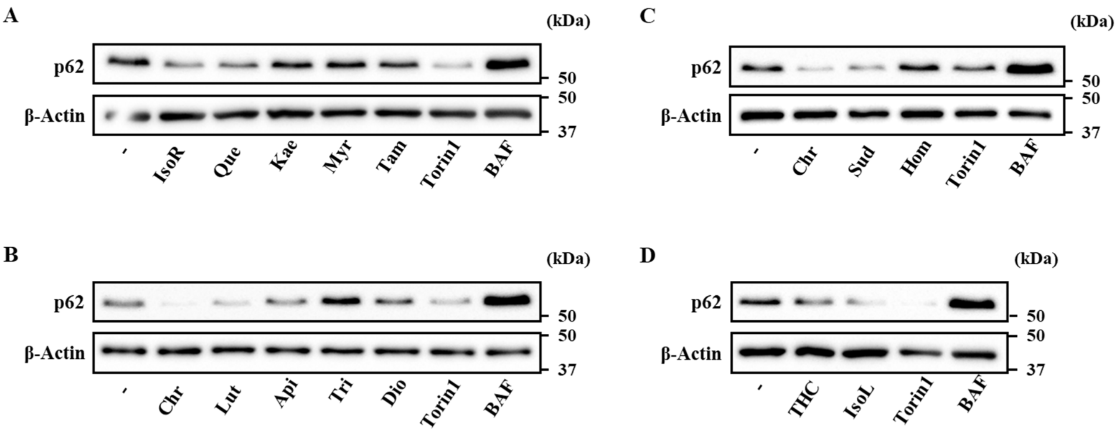

Figure S1. Effects of flavonoids on p62 protein expression in HeLa cells. HeLa cells were treated with each flavonoid (20 μM), Torin1 (250 nM), or bafilomycin A1 (BAF; 100 nM) for 12 h. The level of p62 protein was assessed by western blotting. β-actin was used as an internal control (A-D). IsoR, isorhamnetin; Que, quercetin; Kae, kaempferol; Myr, myricetin; Tam, tamarixetin: Chr, Chrysoeriol; Lut, luteolin; Api, apigenin; Tri, tricetin; Dio, diosmetin; Sud, sudachitin; Hom, homoeriodictyol; THC, 2,2′,4′-trihydroxychalcone; IsoL, isoliquiritigenin.
